# Trends and inequalities in advice and guidance versus direct referral in NHS primary care, 2015-23: population based study

**DOI:** 10.1136/bmjmed-2025-002201

**Published:** 2026-03-18

**Authors:** Kayleigh J Mason, Kelvin P Jordan, James Bailey, Ram Bajpai, Lorna E Clarson, Alice Faux-Nightingale, Tina Hadley-Barrows, John K Haines, Rosie Harrison, Toby Helliwell, Samantha L Hider, Clare Jinks, Natalie Knight, Christian David Mallen, Victoria K Welsh, Claire Louise Burton

**Affiliations:** 1 School of Medicine, Keele University Faculty of Medicine and Health Sciences, Keele, UK; 2 Haywood Academic Rheumatology Centre, Midlands Partnership University NHS Foundation Trust, Haywood Hospital, Stoke-on-Trent, UK; 3 Primary Care and Public Health, Brighton and Sussex Medical School (BSMS), University of Brighton, Falmer, UK

**Keywords:** Primary health care, Healthcare Disparities, Epidemiology, Health policy

## Abstract

**Objectives:**

To examine trends and variation in the use of advice and guidance compared with direct referrals in primary care, and to assess potential inequalities across population groups.

**Design:**

Population based study.

**Setting:**

Clinical Practice Research Datalink (CPRD) Aurum, an anonymised UK primary care database, 1 January 2015 to 31 December 2023.

**Participants:**

16 340 696 patients with electronic health record data registered in CPRD; 671 894 (4% of the registered population) had advice and guidance recorded and 9 719 796 (59% of the registered population) had a direct referral recorded.

**Main outcome measures:**

Annual prevalence of advice and guidance, and direct referrals, in each calendar year, grouped by age, sex, social deprivation, locality, and ethnic group. Mapping of clinical codes to determine target specialities for advice and guidance. Proportion of individuals recorded with advice and guidance and a direct referral within ±4 months.

**Results:**

16 340 696 registered patients were analysed between 2015 and 2023; 671 894 patients (4%) had advice and guidance recorded and 9.7 million (59%) had a direct referral. Use of advice and guidance increased 19-fold from 0.10% to 1.97% of the registered population, doubling between 2019 and 2020 during the covid-19 pandemic. Direct referral rates decreased from 23-25% before the pandemic to 18% in 2020, before recovering to 24% by 2023. Cardiology (21%), dermatology (7%) and ear, nose, and throat (5%) were the most common specialties linked to advice and guidance. Most patients receiving advice and guidance (86%) also had a direct referral within ±4 months. Inequities were evident: use of advice and guidance was higher among older, white, and less deprived patients, whereas minority ethnic and more deprived groups had slower recovery of direct referral rates after the pandemic.

**Conclusions:**

The study showed that the use of advice and guidance has increased substantially since 2015, accelerated by the covid-19 pandemic and maintained after, but has not displaced direct referrals. Direct referral often preceded advice and guidance, raising questions about efficiency and equity. Use of advice and guidance was higher among older, white, and less deprived individuals, whereas minority ethnic and more socially deprived groups were more likely to have direct referrals after advice and guidance, suggesting potential delays in access to specialist care. Policy should prioritise dealing with these inequalities and evaluate whether advice and guidance reduces unnecessary referrals or delays access to specialist care.

**WHAT IS ALREADY KNOWN ON THIS TOPIC**

- Advice and guidance was introduced to support outpatient reform and manage NHS waiting lists
- Evidence on the usage patterns and equity of advice and guidance across patient groups is limited

**WHAT THIS STUDY ADDS**

- Use of advice and guidance increased from 0.1% to 1.97% of the registered population having ≥1 advice and guidance record in a calendar year between 2015 and 2023, particularly after the covid-19 pandemic
- Inequalities were identified, with higher uptake in older, white, and less deprived patients
- Direct referrals recovered to levels before the pandemic, suggesting that advice and guidance is not replacing traditional pathways

**HOW THIS STUDY MIGHT AFFECT RESEARCH, PRACTICE, OR POLICY**

- Future research directions should include looking at the acceptability of advice and guidance to patients and healthcare providers and determining the effect on patient and system outcomes across sociodemographic groups and localities
- Monitoring of equity and targeted support for practices in socially deprived areas should be encouraged, recognising that advice and guidance often occurs together with direct referral and should facilitate community management without prolonging pathways
- The study might inform NHS outpatient transformation and referral optimisation plans by highlighting the increasing advice and guidance that supplements rather than replaces referrals, the potential inequalities in access, and the need for further evaluation and resourcing of services

## Introduction

 Referral is an important part of a primary care clinician's role and describes a process that has a direct consequence on the patient experience of care as well as costs to the healthcare system. Referrals from primary care to secondary or specialist care may be made to establish a diagnosis, start specialist management, access an investigation not available in primary care, or seek a second opinion. Referral usually involves a transfer of clinical responsibility and can be a complex area of decision making, balancing the role of the doctor as both patient advocate and NHS gatekeeper.^1^,^3^

In the UK NHS, referrals for elective (non-emergency) specialist care are usually made through the Choose and Book service (through the electronic referral service), where the options include advice and guidance or refer/advice. Refer/advice allows the user to make a direct referral to a particular specialty.^4^ Established in 2015, advice and guidance is a two way dialogue delivered synchronously (voice call) or asynchronously (electronic communication) that enables primary care to seek specialist input into a patient's care through a formal recorded means. These two elective referral options are the focus of this study.

The use of advice and guidance has been identified as a crucial part of NHS outpatient transformation to deal with the elective care backlog,^5 6^ with the potential to shorten patient journeys through the system,^7 8^ allow prompt appropriate management of conditions requiring specialist advice,^9^ and reduce the burden on primary care that is exacerbated by long waitlists for specialist opinions.^10^ The literature suggests that advice and guidance can be efficient and effective in particular settings, but key aspects for service development include an understanding of suitable patients, equitable access, collaborative working across the health system, consideration of resource implications, and that existing advice and guidance services may still result in a high number of outpatient appointments with relatively few requests ending with expedited patient journeys (eg, investigation requests and direct advice).^411^,^13^

Which patient groups currently use advice and guidance is unclear, however, as is whether advice and guidance is an adjunct to direct referral. The order of elective care requests (eg, advice and guidance before, at the time of, or after direct referral) and whether variation exists in the use of advice and guidance by sociodemographic characteristics are also unclear. Although the use of advice and guidance has been identified as a crucial part of managing NHS waiting lists, little evidence exists to understand its effectiveness in reducing compound pressures while ensuring equity of use. In this national study, in UK primary care, we sought to determine the use of advice and guidance over time compared with direct referrals, variations in advice and guidance by sociodemographic characteristics, rates of advice and guidance by specialty, and the percentage of advice and guidance that ended with a direct referral.

## Materials and methods

### Study design and setting

Electronic health record data were sourced from Clinical Practice Research Datalink (CPRD) Aurum, an anonymised UK primary care database covering 16.2 million individuals (about 24% of the UK population) from 1784 current and historic general practices, mainly in England.^14^ Use of linked data for patient information on index of multiple deprivation and CPRD Aurum Ethnicity Record provided representative distributions across deprivation and ethnic groups compared with national data on the UK population.^15 16^ The study was approved by the CPRD Research Data Governance and used the June 2024 data release.^9^ The approved protocol was made available to the reviewers of this manuscript.

### Study population

All individuals, regardless of age, with advice and guidance or direct referral coded in their primary care record between 1 January 2015 and 31 December 2023 comprised the numerator population. Code lists for advice and guidance, and direct referrals are available online (https://doi.org/10.21252/t55f-vr93). Fast track referral for suspected cancer and emergency referrals, or referral with an intention for admission to hospital, were excluded. The denominator population was the total registered population at the start of each calendar year.

### Covariates

Covariates included age at date of advice and guidance request or referral, gender (as recorded in CPRD), geographical region, ethnic group based on the CPRD derived ethnic group algorithm,^15^ neighbourhood deprivation (index of multiple deprivation 2019 linked to patient postcode) categorised into five equal groups, and Clinical Commissioning Group pseudonym. Clinical Commissioning Groups were clinically led organisations within the NHS that superseded primary care trusts in planning, assessing, and purchasing healthcare services for 106 localities across England,^17^ and were superseded by Integrated Care Boards on 1 July 2022. Given the use of both Clinical Commissioning Group and Integrated Care Board localities during the study timeframe, we have used the term commissioning localities.

### Mapping advice and guidance records with symptom or diagnosis codes

A mapping exercise allowed target specialities to be determined with coded events in the 14 days before and on the date of an advice and guidance record. The mapping exercise was restricted to advice and guidance, and direct referrals recorded in 2023, because 2023 was the most recently available year with the greatest number of advice and guidance records available. Based on a random sample of 500 000 individuals with direct referral recorded in 2023 (categorised to electronic referral specialties, https://doi.org/10.21252/t55f-vr93),^18^ the most common symptom or diagnosis codes recorded 0-14 days before a direct referral were matched to symptom or diagnosis codes recorded 0-14 days before advice and guidance where specialty was not recorded. Likely specialties were assigned to advice and guidance records based on the closest recorded code, with the highest frequency specialty recorded for that code. Several of the authors who were doctors (CLB, TH, and VKW) reviewed the most frequent direct referral specialties and excluded referrals to physiotherapy, private practitioners, health promotion, occupational health, speech and language therapy, or orthotics and prosthetics as likely specialties or services for advice and guidance.

### Prevalence of direct referrals compared with electronic referral service data dashboard

To validate referral rates from CPRD Aurum, referral data recorded between 30 December 2019 and 8 September 2024 were obtained from the NHS electronic referral service open data dashboard.^18^ Referrals were included in annual totals if categorised as routine or urgent and excluded if categorised as two week wait or cancer referrals, or both. Annual mid-year population estimates for England for 2020-23 were obtained from the Office for National Statistics and used as the denominator population to calculate rates for the electronic referral service data.^19^

### Statistical analysis

We modelled annual person level prevalence (ie, ≥1 advice and guidance or referral, or both, per year) to avoid double counting request or response entries and to minimise heterogeneity in practice level coding. Annual person level prevalence for advice and guidance, direct referrals, and NHS electronic referral service open data dashboard were calculated as a percentage of the registered population having ≥1 advice and guidance (or referral, as appropriate) recorded during the year. Trends for advice and guidance, and direct referrals were analysed overall and grouped by age, sex, index of deprivation group, ethnic group, geographical region, and commissioning localities to assess variations over time.

We calculated the percentage of individuals recorded with advice and guidance who also had a record of a direct referral in the four months (122 days; agreed by clinical consensus) before (ie, −122 to 0 days) and after (ie, 1-122 days) the advice and guidance record. This percentage of individuals was compared by the covariates (excluding those with <4 months of follow-up) descriptively and by using multinomial logistic regression. Hence we compared those who received advice and guidance only with those with a direct referral recorded before the advice and guidance and those recorded with a direct referral after the advice and guidance (relative risk ratio and adjusted relative risk ratio with 95% confidence intervals). Age, sex, region, ethnic group, and index of deprivation group were included as covariates in the adjusted models. We also performed a sensitivity analysis for 12 months before and after the advice and guidance record (excluding those with <12 months of follow-up). All models included robust standard errors clustered at the practice level.

Absence of a code for advice and guidance or direct referral was assumed to indicate no consultation having occurred. All included individuals had year of birth (to calculate age), consultation year, and sex recorded. Individuals with no data for ethnic group were recorded as unknown, and as missing for index of deprivation group. Data management and statistical analyses were performed with Stata MP version 19.5 (StataCorp, USA). This study met all five of the CODE-EHR (context, ontology, data, ethics, and rights-electronic health record) minimum framework standards for the use of structured healthcare data in clinical research, with one preferred criterion also met (ethics and governance). Full details are provided in the completed CODE-EHR checklist (online supplemental table S1).^20^

### Patient and public involvement

Patients and the public were involved at all stages of the study. A public contributor (JKH) was a co-applicant on the grant and attended monthly study meetings. Our dedicated patient and public involvement group advised on study design, ensuring that our focus on inequalities in the use of advice and guidance reflected patient priorities. The group highlighted concerns, such as digital literacy and access to transport, which informed our interpretation of inequities. Members of the patient and public involvement group reviewed the findings and contributed to the framing of recommendations about efficiency and equity. The group met every three months to ensure members remained connected with the study and could provide insight and input on a regular basis and will support dissemination. Our patient and public partners are codesigning outputs, resources and recommendations for advice and guidance, and have contributed to framework content on a published paper,^21^ suggested questions and activities for community conversations, and will contribute to developing and refining public information materials. We recognise that our patient and public involvement group was not fully representative of all ethnic and socioeconomic backgrounds affected by inequalities, and future work will prioritise broader inclusion.

## Results

We analysed data on 16.3 million registered patients who were included in the annual denominator population at any point during the study period. Between 2015 and 2023, 4.1% (n=671 894) had advice and guidance recorded and 59.5% (n=9 719 796) had a direct referral recorded (table 1). Advice and guidance was more commonly recorded in older patients and women, with direct referrals recorded more commonly in men. Both elective care pathways were more commonly recorded for white patients and for the least deprived individuals (table 1).

**Table 1:** Baseline personal and socioeconomic characteristics of the study population of 16.3 million registered patients, 2015-23

Characteristics	Registered population (No of patients)	Prevalent elective referral record	
		Advice and guidance	Direct referral
Total No of patients	16 340 696	671 894 (4.1)	9 719 796 (59.5)
Sex:			
Women	8 210 533	395 228 (4.8)	5 226 933 (64.3)
Men	8 130 163	276 666 (3.4)	4 492 863 (54.7)
Mean (SD) age (years)	37.9 (23.0)	52.2 (23.0)	43.8 (23.6)
Ethnic group:			
Asian	1 613 764	59 332 (3.7)	827 669 (51.3)
Black	793 256	28 114 (3.5)	427 516 (53.9)
Mixed or multiple	359 394	10 384 (2.9)	182 285 (50.7)
White	12 766 069	563 021 (4.4)	8 050 919 (63.1)
Other	125 268	1680 (1.3)	38 906 (31.1)
Unknown	682 945	9363 (1.4)	192 501 (28.2)
Index of deprivation group:			
Group 1 (least deprived)	3 190 217	142 620 (4.5)	2 007 242 (62.9)
Group 2	3 241 056	141 937 (4.4)	1 972 154 (60.8)
Group 3	3 196 481	137 123 (4.3)	1 883 343 (58.9)
Group 4	3 465 205	135 883 (3.9)	1 973 817 (57.0)
Group 5 (most deprived)	3 204 201	113 906 (3.6)	1 879 441 (58.7)
Missing	43 536	425 (1.0)	3799 (8.7)
Year:			
2015	9 669 741	11 011 (0.1)	2 380 210 (24.6)
2016	9 860 778	13 533 (0.1)	2 376 248 (24.1)
2017	10 150 263	18 761 (0.2)	2 349 400 (23.1)
2018	10 340 988	37 901 (0.4)	2 425 476 (23.4)
2019	10 351 890	48 561 (0.5)	2 465 365 (23.8)
2020	10 454 998	100 067 (1.0)	1 859 484 (17.8)
2021	10 665 333	146 618 (1.4)	2 252 689 (21.1)
2022	10 404 414	168 812 (1.6)	2 376 486 (22.8)
2023	10 302 006	204 696 (2.0)	2 437 414 (23.6)

Data are number (%) unless indicated otherwise.

SD, standard deviation.

### Annual prevalence of advice and guidance, and direct referrals

**Figure 1:**
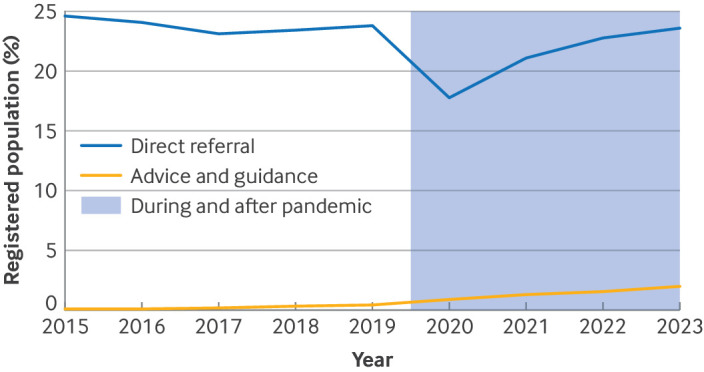
Trends in advice and guidance, and direct referrals, 2015-23. Annual prevalence rates for advice and guidance and direct referrals, showing the rise in use of advice and guidance and the temporary fall in referrals during the covid-19 pandemic

Use of advice and guidance increased from 0.1% in 2015 to 1.97% of the registered population in 2023, with a steep rise during the covid-19 pandemic (figure 1). Direct referrals decreased from about 24% before the covid-19 pandemic to 18% in 2020, before recovering to 24% by 2023 (figure 1). The ratio of direct referral prevalence to advice and guidance prevalence decreased from 235:1 in 2015 to 12:1 in 2023.

### Rates of advice and guidance by socioeconomic factors

**Figure 2:**
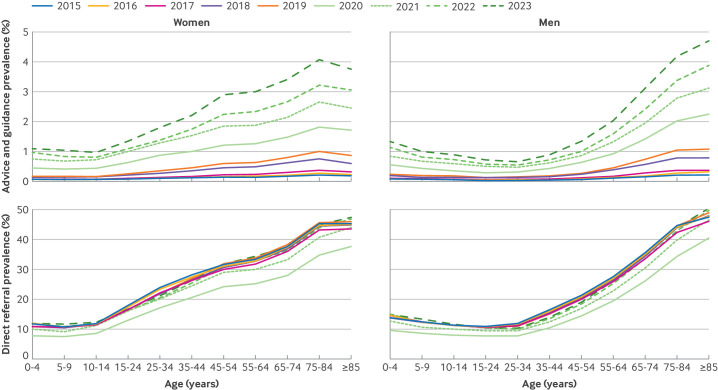
Variation in use of advice and guidance, and referral by age and sex, 2015-23. Distribution of advice and guidance, and direct referrals, across age groups and sex over time

Figure 2 depicts trends in the prevalence of advice and guidance, and direct referrals over time, grouped by age and sex. Online supplemental figure S1 shows the trends grouped by ethnic group and deprivation level. Advice and guidance was more prevalent in women over time, but increased with age for both sexes from age 25 years, peaking in elderly people (75-84 years; figure 2). We saw a steep rise from 2020 to 2023, particularly in older adults (online supplemental table S2).

The prevalence of advice and guidance was higher in white individuals than in Asian, black, and mixed or multiple ethnic groups (online supplemental figure S1). Rates of advice and guidance doubled between 2019 and 2020 for all ethnic groups, likely because of the covid-19 pandemic. We saw inequality in the use of this elective care pathway, with a higher prevalence of advice and guidance in less deprived groups across all ethnic groups from 2020 onwards (online supplemental table S2).

### Rates of direct referral by socioeconomic factors

Direct referrals increased with age from 15 years in women and from 25 years in men, peaking at ages 75-84 years, with a slightly higher referral prevalence in women than in men for most age groups (figure 2). Prevalence rates of direct referrals decreased in 2020 (figure 2), with referral rates increasing in each following year to levels before the covid-19 pandemic by 2023 (online supplemental table S3).

Direct referrals decreased with increasing deprivation across ethnic groups in the years preceding the covid-19 pandemic (2015-19) and after the pandemic (2022-23; online supplemental figure S1). White patients had the highest prevalence for referral, with the mixed or multiple ethnic group having the lowest rates. In 2020-21 (online supplemental figure S1), referral rates decreased substantially, particularly among the more socially deprived and ethnic minority groups (online supplemental table S3).

### Recent variation in rates of advice and guidance, and direct referrals by covariates

**Figure 3:**
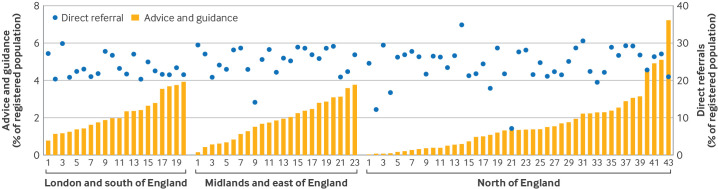
Variation in use of advice and guidance, and direct referrals, across commissioning localities in 2023. Geographical variation in prevalence of advice and guidance and direct referrals showed no consistent relation between greater use of advice and guidance and reduced referral rates. Commissioning localities within each region are numbered from smallest to largest percentage of individuals recorded with advice and guidance in that locality

Focusing on 2023 (because of the highest prevalence of advice and guidance, and recovery of direct referral rates to levels before the covid-19 pandemic), we found a greater prevalence of advice and guidance, and direct referrals for men, older adults, white patients, and less deprived individuals (table 2). We saw wide variation for use of advice and guidance by commissioning locality within each region of England, particularly in the north of England (figure 3 and online supplemental table S4). Greater use of advice and guidance, however, was not associated with lower direct referral rates (correlation coefficient=0.01, P=0.93), indicating that use of advice and guidance did not necessarily result in reduced direct referral (online supplemental table S4).

**Table 2:** Annual prevalence of advice and guidance, and direct referrals as a percentage of the registered population in 2023

Characteristics	Prevalence (% (95% CI))	
	Advice and guidance	Direct referrals
All patients	1.97 (1.96 to 1.98)	23.6 (23.6 to 23.6)
Sex:		
Women	2.40 (2.38 to 2.41)	27.6 (27.6 to 27.7)
Men	1.56 (1.55 to 1.57)	19.7 (19.7 to 19.7)
Age (years):		
0-4	1.22 (1.17 to 1.27)	13.4 (13.3 to 13.6)
5-9	1.03 (1.01 to 1.06)	12.5 (12.4 to 12.6)
10-14	0.93 (0.91 to 0.96)	11.9 (11.8 to 12.0)
15-24	1.02 (1.01 to 1.04)	13.5 (13.4 to 13.5)
25-34	1.18 (1.17 to 1.20)	15.4 (15.4 to 15.5)
35-44	1.52 (1.50 to 1.54)	19.8 (19.7 to 19.8)
45-54	2.08 (2.06 to 2.11)	25.1 (25.0 to 25.1)
55-64	2.50 (2.48 to 2.53)	30.0 (30.0 to 30.1)
65-74	3.23 (3.19 to 3.26)	36.0 (35.9 to 36.1)
75-84	4.12 (4.07 to 4.17)	44.5 (44.3 to 44.6)
≥85	4.12 (4.05 to 4.20)	48.5 (48.3 to 48.8)
Ethnic group:		
Asian	1.85 (1.82 to 1.87)	20.4 (20.4 to 20.5)
Black	1.76 (1.72 to 1.79)	21.4 (21.2 to 21.5)
Mixed or multiple	1.42 (1.37 to 1.47)	17.6 (17.4 to 17.8)
White	2.10 (2.09 to 2.11)	25.1 (25.1 to 25.2)
Other	0.66 (0.61 to 0.72)	9.8 (9.6 to 10.0)
Unknown	0.68 (0.65 to 0.71)	9.6 (9.5 to 9.7)
Index of deprivation group:		
Group 1 (least deprived)	2.15 (2.13 to 2.17)	24.8 (24.8 to 24.9)
Group 2	2.07 (2.05 to 2.09)	24.3 (24.2 to 24.3)
Group 3	2.04 (2.02 to 2.06)	23.5 (23.4 to 23.6)
Group 4	1.88 (1.86 to 1.89)	22.7 (22.7 to 22.8)
Group 5 (most deprived)	1.75 (1.73 to 1.77)	23.0 (22.9-23.1)
Missing	0.62 (0.53 to 0.73)	—
Region:		
London	2.16 (2.14 to 2.17)	22.4 (22.3 to 22.4)
Midlands and east of England	2.09 (2.07 to 2.10)	24.4 (24.3 to 24.4)
North of England	1.32 (1.30 to 1.33)	24.1 (24.1 to 24.2)
South of England	2.29 (2.27 to 2.30)	23.5 (23.4 to 23.5)

CI, confidence interval.

### Proportion of individuals with advice and guidance, and direct referrals recorded

Among patients with a record for advice and guidance, 86% also had a direct referral within ±4 months (table 3). Direct referral more often preceded (69%) than followed (17%) advice and guidance, indicating that advice and guidance was frequently used after referral rather than as a substitute or precursor (table 3). The proportion of individuals with advice and guidance only was marginally higher in 2015-17 (17-18%) before stabilising at 13-14% in more recent years (2018-23; table 3). This finding challenges the assumption that advice and guidance typically precedes referral. Inequalities were evident: advice and guidance was more commonly used by older, white, and less socially deprived patients, whereas minority ethnic groups were more likely to have direct referral after advice and guidance, suggesting potential delays in access to specialist care for these populations (table 3). These patterns persisted in sensitivity analyses based on a window of ±12 months rather than ±4 months (online supplemental table S5).

**Table 3:** Association between advice and guidance, and direct referral within ±4 months. Proportion of patients with both advice and guidance, and direct referral within ±4 months, grouped by personal and socioeconomic factors

Characteristics	Advice and guidance only (No (%))	Direct referral*		Before advice and guidance		After advice and guidance	
		Before advice and guidance (No (%))	After advice and guidance (No (%))	Relative risk ratio (95% CI)	Adjusted relative risk ratio (95% CI)	Relative risk ratio (95% CI)	Adjusted relative risk ratio (95% CI)
No of patients	81 861 (14)	413 595 (69)	103 854 (17)	—	—	—	—
Sex:							
Women	47 061 (13)	245 269 (69)	61 227 (17)	Reference	Reference	Reference	Reference
Men	34 800 (14)	168 326 (68)	42 627 (17)	0.93 (0.91 to 0.95)	0.93 (0.91 to 0.95)	0.94 (0.92 to 0.96)	0.94 (0.92 to 0.96)
Age (years):							
0-4	1871 (17)	7236 (66)	1844 (17)	0.77 (0.69 to 0.87)	0.80 (0.71 to 0.89)	0.79 (0.70 to 0.89)	0.78 (0.70 to 0.87)
5-9	3406 (17)	13 605 (67)	3403 (17)	0.80 (0.73 to 0.87)	0.81 (0.74 to 0.89)	0.80 (0.74 to 0.87)	0.81 (0.75 to 0.88)
10-14	3338 (16)	13 660 (67)	3304 (16)	0.82 (0.75 to 0.89)	0.82 (0.76 to 0.90)	0.79 (0.73 to 0.86)	0.80 (0.74 to 0.87)
15-24	5967 (16)	25 277 (67)	6570 (17)	0.85 (0.78 to 0.92)	0.85 (0.78 to 0.92)	0.88 (0.83 to 0.93)	0.90 (0.85 to 0.95)
25-34	7406 (14)	35 494 (69)	8828 (17)	0.96 (0.92 to 1.00)	0.96 (0.92 to 1.00)	0.96 (0.91 to 1.00)	0.96 (0.92 to 1.01)
35-44	9559 (14)	47 792 (69)	11 927 (17)	Reference	Reference	Reference	Reference
45-54	11 756 (13)	60 422 (69)	15 341 (18)	1.03 (0.99 to 1.07)	1.04 (1.00 to 1.08)	1.05 (1.00 to 1.09)	1.07 (1.02 to 1.11)
55-64	12 794 (13)	67 218 (69)	17 030 (18)	1.05 (1.00 to 1.10)	1.07 (1.02 to 1.12)	1.07 (1.02 to 1.12)	1.12 (1.07 to 1.17)
65-74	12 460 (13)	64 368 (69)	16 508 (18)	1.03 (0.97 to 1.10)	1.07 (1.01 to 1.13)	1.06 (1.00 to 1.13)	1.12 (1.06 to 1.18)
75-84	9550 (12)	56 147 (71)	13 792 (17)	1.18 (1.08 to 1.28)	1.21 (1.13 to 1.30)	1.16 (1.08 to 1.24)	1.23 (1.16 to 1.31)
≥85	3754 (12)	22 376 (71)	5307 (17)	1.19 (1.09 to 1.31)	1.23 (1.13 to 1.33)	1.13 (1.04 to 1.23)	1.20 (1.11 to 1.30)
Ethnic group:							
Asian	6897 (13)	34 329 (64)	12 040 (23)	0.99 (0.81 to 1.22)	1.01 (0.83 to 1.23)	1.44 (1.23 to 1.69)	1.55 (1.34 to 1.80)
Black	2978 (12)	17 702 (70)	4503 (18)	1.18 (0.91 to 1.54)	1.16 (0.92 to 1.48)	1.25 (1.03 to 1.52)	1.35 (1.14 to 1.61)
Mixed or multiple	1231 (13)	6281 (68)	1709 (19)	1.02 (0.88 to 1.17)	1.09 (0.95 to 1.25)	1.15 (1.03 to 1.29)	1.32 (1.18 to 1.47)
White	69 455 (14)	348 488 (69)	83 941 (17)	Reference	Reference	Reference	Reference
Other	185 (13)	989 (68)	290 (20)	1.07 (0.84 to 1.36)	1.13 (0.89 to 1.43)	1.30 (1.03 to 1.63)	1.47 (1.17 to 1.83)
Unknown	1115 (13)	5806 (70)	1371 (17)	1.04 (0.84 to 1.29)	1.09 (0.89 to 1.35)	1.02 (0.79 to 1.31)	1.09 (0.84 to 1.40)
Index of multiple deprivation group:							
Group 1 (least deprived)	16 525 (13)	87 789 (69)	22 619 (18)	Reference	Reference	Reference	Reference
Group 2	18 730 (15)	85 162 (67)	22 842 (18)	0.86 (0.72 to 1.02)	0.86 (0.73 to 1.03)	0.89 (0.80 to 0.99)	0.89 (0.80 to 0.99)
Group 3	17 244 (14)	83 128 (68)	22 554 (18)	0.91 (0.74 to 1.12)	0.92 (0.75 to 1.13)	0.96 (0.83 to 1.10)	0.93 (0.81 to 1.07)
Group 4	16 041 (13)	84 166 (69)	21 202 (17)	0.99 (0.76 to 1.29)	1.00 (0.77 to 1.30)	0.97 (0.80 to 1.17)	0.93 (0.77 to 1.11)
Group 5 (most deprived)	13 245 (13)	73 173 (72)	14 529 (14)	1.04 (0.79 to 1.37)	1.06 (0.81 to 1.39)	0.80 (0.67 to 0.96)	0.78 (0.66 to 0.93)
Missing	76 (21)	177 (49)	108 (30)	0.44 (0.22 to 0.88)	0.43 (0.21 to 0.89)	1.04 (0.56 to 1.93)	1.10 (0.57 to 2.12)
Year:							
2015	1653 (17)	6337 (63)	1993 (20)	0.70 (0.50 to 0.98)	0.72 (0.51 to 1.00)	1.33 (0.97 to 1.83)	1.39 (1.01 to 1.90)
2016	2070 (17)	7666 (62)	2538 (21)	0.68 (0.50 to 0.93)	0.69 (0.50 to 0.94)	1.36 (0.94 to 1.95)	1.39 (0.97 to 1.98)
2017	3048 (18)	10 391 (62)	3384 (20)	0.63 (0.44 to 0.89)	0.63 (0.44 to 0.90)	1.23 (0.86 to 1.75)	1.27 (0.89 to 1.79)
2018	4364 (13)	23 736 (70)	5678 (17)	1.00 (0.75 to 1.33)	1.00 (0.75 to 1.34)	1.44 (1.17 to 1.77)	1.47 (1.20 to 1.80)
2019	5920 (14)	27 409 (64)	9176 (22)	0.85 (0.69 to 1.04)	0.85 (0.69 to 1.04)	1.71 (1.48 to 1.99)	1.75 (1.51 to 2.02)
2020	11 978 (14)	58 627 (66)	18 033 (20)	0.90 (0.78 to 1.03)	0.90 (0.78 to 1.04)	1.67 (1.47 to 1.88)	1.67 (1.48 to 1.89)
2021	16 069 (13)	82 782 (68)	23 682 (19)	0.95 (0.85 to 1.06)	0.95 (0.85 to 1.06)	1.63 (1.43 to 1.86)	1.64 (1.44 to 1.86)
2022	17 918 (13)	93 936 (70)	22 335 (17)	0.96 (0.89 to 1.04)	0.97 (0.89 to 1.05)	1.38 (1.23 to 1.54)	1.38 (1.24 to 1.55)
2023	18 841 (14)	102 711 (74)	17 035 (12)	Reference	Reference	Reference	Reference

* Before advice and guidance defined as direct referral recorded on −122 to 0 days; after advice and guidance defined as direct referral recorded on 1-122 days.

CI, confidence interval.

### Mapping

**Figure 4:**
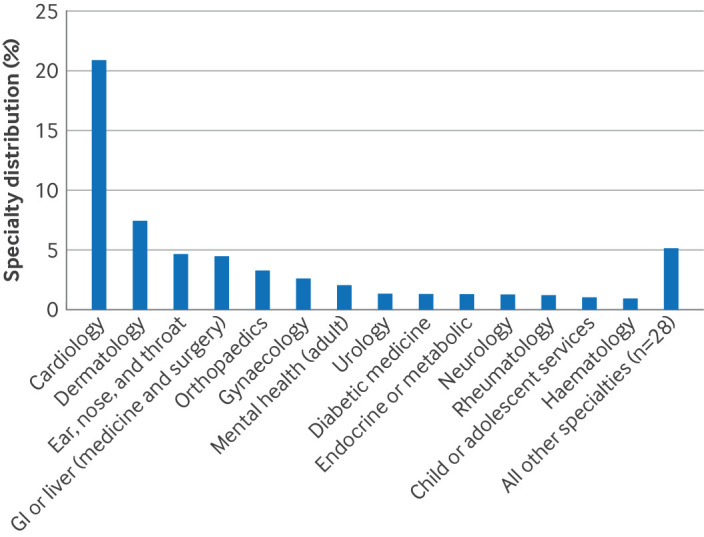
Specialty distribution of advice and guidance requests after mapping data for 2023. Most common specialties recorded within 14 days of an advice and guidance entry, after mapping to consultation codes for direct referrals. GI=gastrointestinal

Of 162 787 individuals with their first advice and guidance record in 2023, 2% had a specialty recorded in 0-14 days before the advice and guidance. Matching consultation codes recorded for direct referrals resulted in an additional 59% (61% total) attributed to a specialty. Cardiology, dermatology, and ear, nose, and throat were the most frequent specialties (figure 4).

### Comparison with electronic referral service dashboard data

Rates of direct referral in CPRD Aurum were comparable with rates calculated based on data from the NHS electronic referral service open data dashboard (online supplemental table S6).

## Discussion

### Principal findings

In this study, we saw an increase from 0.89% to 1.97% of the registered population in the use of advice and guidance during and after the covid-19 pandemic (2020-23), representing a systematic shift in how specialist input is accessed.^6^ This increase did not displace direct referrals, however, which returned to levels before the pandemic by 2023. Most patients receiving advice and guidance were also referred within four months, suggesting that the service often adds a step rather than substitutes for referral.

Inequities were clear: advice and guidance was more often used for older, white, and less deprived patients, whereas minority ethnic and more socially deprived groups showed slower recovery of direct referral access after the pandemic. At the locality level, patterns varied widely, and greater use of advice and guidance was not associated with lower referral rates, raising questions about the efficiency of the pathway and whether it genuinely empowers doctors to manage more care in the community.^5^

### Comparison with other studies

Our findings align with national policy initiatives during the covid-19 pandemic,^6^ which promoted advice and guidance to maintain specialist input while reducing face-to-face outpatient activity and attempting to reduce pressures on the elective healthcare system in the pandemic recovery period. NHS England's elective care recovery plan aimed to deliver 30% more elective activity by 2024-25 compared with levels before the pandemic (2019-20), partly through wider use of advice and guidance.^22^ Although we could not calculate advice and guidance to outpatient ratios, our results suggested substantial progress towards this target.

A scoping review defined four domains of referral quality: clinical information, clinical reasoning, patient factors, and system barriers.^21^ Quality improved with the use of electronic templates, clear referral questions, guideline adherence, appropriate urgency, and recorded preferences, whereas common deficits included missing histories or examinations, and weak feedback loops. Exemplar evaluations of local specialty advice and guidance implementation are described.

1. Advice and guidance embedded in the NHS electronic referral service since 2017 supported about 6500 patients' referral to neurology in Newcastle-upon-Tyne over 2.5 years, with a practical model for set-up, turnaround management, and selection of a condition.^8^ The authors reported efficiencies in triage and outlined barriers and solutions that other sites have since adopted.
2. In an evaluation of >2000 patients for general surgery in Birmingham, advice and guidance replies were typically same day with a substantial reduction in new outpatient appointments (16%) after advice and guidance was introduced, indicating improved triage and pre-clinic work-up.^10^
3. National guidelines were published by NHS England's Referral Optimisation for Skin Conditions and the British Association of Dermatologists’ to answer frequently asked questions from the NHS electronic referral service, and advice and guidance. The guidelines described tele-dermatology enabled advice and guidance with rapid turnaround and image attachments to streamline referrals and mitigate inequities in access for inflammatory skin disease.^23^

Persistence of the relatively high onward referral rate, however, indicates that advice and guidance may not be fulfilling the intended role as a substitute for direct referral because the two elective care pathways often occurred together. The effect on efficiency and outcomes for patient journeys requires further evaluation.

The socioeconomic and ethnic inequalities that we saw mirror wider evidence indicating that the covid-19 pandemic exacerbated existing health inequalities in England, particularly for socially deprived and minority ethnic groups.^24^ The Health Foundation showed a deprivation gradient of those residing in more deprived areas having poorer access and experience of primary care,^25^ with the NHS Race and Health Observatory reporting higher levels of discrimination and lower trust in primary care by black and Asian patients.^26^ The King's Fund subsequently conducted research into how these inequalities were being dealt with, highlighting barriers such as transport, employment constraints, and lower health literacy.^27^ Our research suggests that inequities arise at the point of referral itself, with advice and guidance disproportionately applied to certain groups before a referral to secondary care is made. Our ongoing qualitative work will provide an in-depth analysis of patient and system factors that influence decision making about mode of referral.

NHS England instructed trusts in 2020 to disaggregate waiting lists by ethnic group and social deprivation, but reducing inequities will require action upstream, including the point of referral. Our findings support calls for more inclusive approaches to outpatient transformation,^7 22^ ensuring that initiatives such as advice and guidance do not unintentionally widen gaps in access. NHS England (2022) published a practical framework for NHS systems to deal with inequalities through actionable insights, which we believe could be implemented to support practices caring for under-resourced or socially deprived populations: start with data (eg, determine practices with greatest inequalities in patients receiving advice and guidance or direct referral); test with lived experience (eg, explore problems in receiving advice and guidance or direct referral for patients most in need); and co-design solutions with patients most in need.^28^

### Strengths and limitations of this study

In this national, population based study, we explored the trends and variations in advice and guidance versus direct referrals from primary care in the NHS. The large dataset and longitudinal design provided a unique opportunity to examine the effect of system-wide changes, including those accelerated by the covid-19 pandemic. Although we did not include outcomes looking at patient or healthcare provider views of advice and guidance, our funded work includes interviews with patients, primary care clinicians, secondary care clinicians, and commissioners. Separate publications are in development to communicate these important perspectives.

Limitations of our study included the potential for misclassification of advice and guidance, and referral coding, although any such errors are likely to be non-differential. We saw low rates of advice and guidance, and direct referral for individuals with an unknown ethnic group. Mathur et al showed that recording of unknown ethnic group was reduced during quality outcomes framework incentivisation (2006-11) and increased again after incentivisation ended.^29^ The authors suggested that those with an unknown ethnic group may correspond to less frequent attenders, and that their ethnic group is not recorded (unlike more frequent attenders). We believe that this suggestion is likely to be reflected in our data, with lower rates of advice and guidance, and direct referral reported in our ethnic groups. Uptake of alternative telemedicine providers may not be consistently coded in electronic health records, meaning some specialist input was unobserved. Differences in clinical coding standards between practices (some only capturing advice and guidance requests or responses rather than the full pathway) and differential use of advice and guidance across the healthcare system affected our ability to infer the true burden of advice and guidance on primary care. Automation of advice and guidance recording in existing electronic health record systems could enhance clinician usability and record keeping. Our analysis was descriptive and cannot establish causality between service design, sociodemographic characteristics, and referral outcomes. Lastly, recent policy changes incentivising advice and guidance coding were too recent to be captured in our data.^30^

### Policy implications

For advice and guidance to support NHS priorities, reducing elective backlogs and moving care into the community, three actions are required*.* The first is efficiency, evaluating whether advice and guidance prevents unnecessary referrals or simply delays referrals, including assessment of patient outcomes and system costs. The second is equity, routinely monitoring advice and guidance by social deprivation and ethnic group, and support practices caring for under-resourced or socially deprived populations to ensure that referral pathways do not reinforce inequalities. The third is primary care capacity, investing in training, coding support, and digital infrastructure for doctors so that advice and guidance enables community management rather than becoming an additional administrative step. Without these measures, advice and guidance risks functioning as a bottleneck in the referral process rather than a tool for transformation aligned to delivery of the NHS 10 year plan.^5^

### Conclusions

Advice and guidance use has risen sharply across the NHS between 2015 and 2023, but no corresponding reduction in direct referrals has been seen over this period. Advice and guidance seem to be layered onto existing pathways, with potential inefficiencies and risks of widening inequities. We found that older, white, and less deprived populations were disproportionately accessing advice and guidance, whereas minority ethnic groups were more likely to have direct referral after advice and guidance, suggesting potential delays in access to specialist care. If advice and guidance is to contribute meaningfully to the NHS long term plan to move care into the community and reduce elective care backlogs,^31^ further evaluation and resourcing are needed to improve efficiency and equity, ensuring advice and guidance contributes to shorter, fairer, and more effective patient pathways.

## Supplementary material

10.1136/bmjmed-2025-002201 online supplemental file 1

## Data Availability

Data may be obtained from a third party and are not publicly available.
