# Supplementary material for: Trends and inequalities in advice and guidance versus direct referral in NHS primary care, 2015-23: population based study
Source: BMJ Med. 2026 Mar 18;5(1):e002201. doi: 10.1136/bmjmed-2025-002201 (PMC13007146; doi:10.1136/bmjmed-2025-002201)
Supplement: Supplementary file 2 [file bmjmed-2025-002201.reviewer_comments.pdf]

PEER REVIEW HISTORY

BMJ Medicine publishes all reviews undertaken for accepted manuscripts. Reviewers are asked to complete a checklist review form and are provided with free text boxes to elaborate on their assessment. These free text comments are reproduced below.

ARTICLE DETAILS

**TITLE (PROVISIONAL)**	Trends and inequalities in Advice and Guidance versus direct referral in NHS primary care: a population-based study, 2015–2023
**AUTHORS**	Mason, Kayleigh; Jordan, Kelvin; Bailey, James; Bajpai, Ram; Clarson, Lorna; Faux-Nightingale, Alice; Hadley-Barrows, Tina; Haines, John; Harrison, Rosie; Helliwell, Toby; Hider, Samantha; Jinks, Clare; Knight, Natalie; Mallen, Christian; Welsh, Victoria; Burton, Claire

VERSION 1 - REVIEW

**REVIEWER NAME**	Nordlund, Niko
**REVIEWER AFFILIATION**	University of Helsinki Faculty of Medicine
**REVIEWER CONFLICT OF INTEREST**	None
**AI DISCLOSURE**	I have used AI (ChatGPT) for grammar and spell check.
**DATE REVIEW RETURNED**	21-Oct-2025

**GENERAL COMMENTS**	This is the first national, population-based study examining trends and inequalities in the use of Advice and Guidance versus direct referrals in the NHS. The topic is timely and relevant to current NHS goals. The work provides important data to inform public policy discussions about equitable access to specialist care and NHS sustainability.<br><br>Abstract<br>Page 4, line 18: The meaning of "4%" is not immediately clear. As it refers to the proportion of the total population, while "59%" refers to a different denominator, the comparison is confusing. Please clarify what each percentage represents.<br><br>Page 4, line 33: The statement "The system appears to benefit older, white, and less deprived individuals" is not fully supported by the data and may contradict the discussion, which highlights the unknown benefit of A&G and its potential to create bottlenecks. Rephrase to indicate that A&G use was higher among these groups, without implying improved patient outcomes.<br><br>Page 4, line 52: The increase in A&G use is described as 19-fold here and as 20-fold in the Results section. Please standardize for consistency.<br><br>Methods<br>Page 6, line 8: Please discuss the representativeness of CPRD Aurum for studying A&G use. How well does the dataset reflect the national population in terms of age, deprivation, and ethnicity?<br><br>Page 8, line 3: The description of patient involvement is positive, but the manuscript does not currently convey patient perspectives. Do

	patients perceive A&G as helpful? Likewise, how do clinicians view it as a tool for patient management? These viewpoints would strengthen the discussion of equity and highlight possible benefits and harms of A&G.<br><br>Results<br>Figure 2: This figure contains substantial information but appears cluttered. Consider simplifying it. Additionally, joint analysis of age and ethnicity should be added, as differences in age structure could partly account for ethnic variations in A&G and referral rates.<br><br>Discussion<br>Page 14, line 12: The discussion appropriately acknowledges that patient-important outcomes were not assessed. However, the later statement calling for a “redesign” of A&G may be too strong given that the study is descriptive and does not assess outcomes or cost-effectiveness. Consider softening this to recommend further evaluation before policy redesign is proposed.

**REVIEWER NAME**	Ismail, Ismail
**REVIEWER AFFILIATION**	King's College London, Department of Population Health Sciences
**REVIEWER CONFLICT OF INTEREST**	None
**AI DISCLOSURE**	No
**DATE REVIEW RETURNED**	21-Oct-2025

**GENERAL COMMENTS**	Thank you for this interesting research on A&G and referral rates. It raises several important points about the variations in direct referral and A&G rates between different ethnic groups, and their trends over the years. With the recent push towards advice and guidance, this paper provides important data on its implementation and equity implications.<br>However in regards to your third aim, I have a couple of questions/comments about the methodology and results.<br><br>Methodology<br>1. How did you handle cases where a symptom could be mapped to several specialties?<br>2. It is unclear whether you only mapped 2023 cases where specialty was unclear, or applied this to all years (2015-2023). Could you clarify the scope of this mapping exercise?<br>3. You mentioned that the mapping algorithm was built using 2023 referral patterns - how did you account for evolving guidelines over the years, particularly if you applied this retrospectively to your study period?<br><br>Results<br>1. Related to the second comment in the methodology subsection - if you did map all the years, then what were the findings for the earlier years? Did the proportion of mappable cases or specialty distribution change over time?<br>2. What happened to the 39% of the cases where a specialty could not be matched for 2023? Did this vary by ethnicity?<br><br>Regarding your discussion<br>1. You stated that this is the first national population study - are there any comparisons to smaller or regional studies which could provide additional context?

	2. You observed disparities but have not discussed possible reasons/mechanisms for this. I feel a brief discussion with existing literature may be useful to help contextualize your findings.<br>3. I do not think stating that A&G adds an additional step of unknown benefit is valid, as additional step seems to imply an unnecessary delay. There might be other reasons why a GP might A&G after referral, such as to ask for management advice whilst the patient waits to see the specialist. Without data on A&G content or patient outcomes, it may not be right to frame it the way as stated in the paper.<br>4. You stated to support practices serving disadvantaged populations - do you have any suggestions on how to do this.

**REVIEWER NAME**	Sutton, Matt
**REVIEWER AFFILIATION**	The University of Manchester
**REVIEWER CONFLICT OF INTEREST**	None
**AI DISCLOSURE**	No
**DATE REVIEW RETURNED**	28-Oct-2025

**GENERAL COMMENTS**	This manuscript uses a large sample of person-level records from the Clinical Practice Research Datalink to examine trends in the recording of ‘advice and guidance’ and ‘referral to specialist’ between 2015 and 2023. They also examine differences across population groups. There is interest in whether the two types of activity are occurring together and, if so, in what order and the paper uncovers some differences in this between population groups.<br><br>I found the paper to be interesting and novel. Some comments that I think need to be addressed are below:<br><br>An easily understandable term should be used for “Advice & Guidance” which appears to be an English NHS term<br><br>The abstract needs a clear statement on why this is interesting. Why does it matter who gets “advice & guidance”? Why does it matter if this comes before or after referral? The text in the Conclusion suggests the authors have some concerns in mind (eg. efficiency, equity) but they don’t say so upfront<br><br>Abstract should contain total population size<br><br>It doesn’t really make sense to talk a 19-fold increase when the baseline rate is so small. It is fine to say 1 in a 1000 people up to 20 in a 1000 people<br><br>This sentence in the introduction is absolutely critical “Established in 2015, A&G is a two-way dialogue delivered synchronously (voice call) or asynchronously (electronic communication) that enables primary care to seek specialist input into a patient’s care through formal recorded means.” This is not at all clear from the label (I assumed it was A&G from the GP to the patient) and ‘remote specialist advice’ or something like that should be used throughout instead.<br><br>No justification is given for analysing whether or not an individual received an A&G or direct referral within a year, rather than the number of A&G or direct referrals within a year

	Lines (38-29, page 5): I don't understand how region and commissioning locality could both be included as covariates. I assume one is nested in the other<br><br>No justification is given for including either region or commissioning locality or both as covariates<br><br>Differences in recording between practices would likely be important but practice is not included as one of the covariates. We therefore do not know whether the differences by population subgroup are due to within- or between-practice differences.<br><br>I was not clear how the denominator population has been defined. The figure given is 16.3million, which sounds like a snapshot for one point in time rather than repeated value for every year. Some patients will join and some will leave the registered population over the period so it is unclear how the prevalent rates in Table 1 are calculated<br><br>This sentence (lines 55-56 page 8) is critical: "This challenges the assumption that A&G typically precedes referral." This needs to come before the analysis is presented to explain why the work is being conducted. It is not obvious to the reader on first reading.<br><br>While the rates of use of A&G and direct referral are interesting, I think the analysis of whether they both happen and, if so, in which order, is a more interesting line of enquiry.

**REVIEWER NAME**	Harron, Katie
**REVIEWER AFFILIATION**	
**REVIEWER CONFLICT OF INTEREST**	None
**AI DISCLOSURE**	No
**DATE REVIEW RETURNED**	19-Nov-2025

**GENERAL COMMENTS**	This is an interesting description of the use of A&G and direct referrals in primary care. I have a few points for clarification.<br><br>Firstly, I think there needs to be a more clear and upfront description of what A&G is, and why it might be used (with and without referral). A clinical example here would be helpful.<br><br>Abstract: please give the total number of individuals/person-years included<br><br>Given the authors are interested in trends in the use of A&G, I think it would make more sense to describe and model the total number of A&G events per person-year, rather than the proportion of patients with at least one A&G event within a given year. What were trends in the total numbers of A&G like?<br><br>How has the number of individuals who were assigned A&G alone, compared with both A&G and direct referral, changed over time?<br><br>The multinomial modelling needs more explanation and description. Was this a single model, with direct referral before / direct referral

	after / no direct referral as the outcome? Or were separate models used?<br><br>How was clustering of observations within practices dealt with?<br><br>Fig2: The text states that "Although A&G is more prevalent in males over time, a higher percentage of females were recorded with A&G due to males experiencing more episodes" I don't understand this explanation. The plot is showing percentages– so relative comparisons shouldn't be affected by the total number of episodes. Table 1 also indicates that prevalence is higher in females (not males).<br><br>The annual prevalences in table 2 are presented differently to the overall prevalence in Table 1, i.e. as a rate rather than a percentage. Have these been calculated differently or do they represent the same thing, but for different periods? Table 2 is labelled as prevalence rate %, which is unclear.<br><br>Table 3- which period is this for? Given the unadjusted and adjusted results are similar, and given there is a lot going on in this table, I suggest keeping one set of results here and putting the rest in an abstract. I think that would make the comparisons (before / after; between demographic groups) easier to digest. The adjusted results I think are adjusted for CCG but the coefficients are not presented. What was variation by CCG like?<br><br>Fig 3 – what do the numbers along the x axis refer to?<br><br>Table S2 etc– need to state what the numbers in the parentheses refer to.

VERSION 1 – AUTHOR RESPONSE

Reviewer: 1 - Mr. Niko Nordlund, University of Helsinki Faculty of Medicine
Comments to the Author

This is the first national, population-based study examining trends and inequalities in the use of Advice and Guidance versus direct referrals in the NHS. The topic is timely and relevant to current NHS goals. The work provides important data to inform public policy discussions about equitable access to specialist care and NHS sustainability.

Abstract

1. Page 4, line 18: The meaning of "4%" is not immediately clear. As it refers to the proportion of the total population, while "59%" refers to a different denominator, the comparison is confusing. Please clarify what each percentage represents.

We agree and have clarified the denominators in the **Abstract**. We now state explicitly that 4% of the registered population had Advice & Guidance (A&G) recorded at least once during 2015–2023, whereas 59% had at least one direct referral recorded in the same period. We have removed the proportion of females in each group.

Abstract (Methods/Outcomes): annual prevalence of A&G and direct referrals was calculated as the percentage of the registered population having ≥1 event in each calendar year, stratified by […]

Abstract (Results): We analysed 16,340,696 registered patients between 2015–2023;~~,~~ 671,894 patients (4%~~; 59% female~~) had A&G recorded and 9.7 million (59%~~; 46% female~~) had a direct referral recorded.

2. Page 4, line 33: The statement "The system appears to benefit older, white, and less deprived individuals" is not fully supported by the data and may contradict the discussion, which highlights the unknown benefit of A&G and its potential to create bottlenecks. Rephrase to indicate that A&G use was higher among these groups, without implying improved patient outcomes.
We have rephrased to avoid implying benefit and instead describe differential use.

Abstract (Conclusion): A&G use was higher among ~~*The system appears to benefit*~~ older, white, and less deprived individuals […]

3. Page 4, line 52: The increase in A&G use is described as 19-fold here and as 20-fold in the Results section. Please standardize for consistency.
We have standardised to percentages in line with comment 4 by reviewer 3 by making changes to the following sentences:

Abstract – *Results* (second sentence): A&G use increased ~~*19-fold*~~ from ~~(~~0.10% to 1.97%~~)~~, […]

What this study adds: A&G use increased ~~*19-fold*~~ from 0.1% to 1.97% between 2015 and 2023, particularly post-pandemic.

Annual Prevalence of A&G and Direct Referrals (first sentence): A&G use rose ~~*almost 20-fold*~~ from 0.1% in 2015 to ~~*2.0%*~~ 1.97% in 2023

Discussion (first sentence): We observed an ~~*almost twenty-fold*~~ increase from 0.89% to 1.97% in the use of A&G during and following the COVID-19 pandemic (2020-2023), […]

Methods
4. Page 6, line 8: Please discuss the representativeness of CPRD Aurum for studying A&G use. How well does the dataset reflect the national population in terms of age, deprivation, and ethnicity?
We have added to the **Methods** summarising representativeness for ethnicity and deprivation recording and cited the validation studies.

Methods - *Study design and setting* (second sentence): Use of linked data for patient-level Index of Multiple Deprivation data and CPRD Aurum Ethnicity Record provides representative distributions across deprivation and ethnic groups compared to national data on the UK population.[15,16]

References:

15. Shiekh SI, Harley M, Ghosh RE, Ashworth M, Myles P, Booth HP, et al. Completeness, agreement, and representativeness of ethnicity recording in the United Kingdom's Clinical Practice Research Datalink (CPRD) and linked Hospital Episode Statistics (HES). Popul Health Metr [Internet]. 2023 Dec 1 [cited 2025 Mar 12];21(1):1–13. Available from: https://pophealthmetrics.biomedcentral.com/articles/10.1186/s12963-023-00302-0

16. Mahadevan P, Harley M, Fordyce S, Hodgson S, Ghosh R, Myles P, et al. Completeness and representativeness of small area socioeconomic data linked with the UK Clinical Practice Research Datalink (CPRD). J Epidemiol Community Health [Internet]. 2022 Oct 1 [cited 2025 Mar 12];76(10):880–6. Available from: https://jech.bmj.com/content/76/10/880

5. Page 8, line 3: The description of patient involvement is positive, but the manuscript does not currently convey patient perspectives. Do patients perceive A&G as helpful? Likewise, how do clinicians view it as a tool for patient management? These viewpoints would strengthen the discussion of equity and highlight possible benefits and harms of A&G.

We recognise that patient and clinician viewpoints are vital for understanding the possible benefits and harms of A&G. However, healthcare data regarding A&G use in databases such as CPRD do not include the viewpoints of patients or clinicians so we are unable to convey these perspectives in our analyses presented here. We have embedded public involvement throughout the study as described in our **Methods** section. The funded work includes an extensive qualitative work package focusing on

the perspectives of patients, primary and secondary care clinicians and healthcare commissioners regarding the use of A&G and its potential benefits and disadvantages. These findings will be published separately.

Discussion – *Strengths and Limitations* (third sentence, first paragraph): While this study did not include outcomes addressing patient or healthcare provider views of A&G, our funded work includes interviews with patients, primary care clinicians, secondary care clinicians, and commissioners. Separate publications are in development to communicate these important perspectives.

Results

6. Figure 2: This figure contains substantial information but appears cluttered. Consider simplifying it. Additionally, joint analysis of age and ethnicity should be added, as differences in age structure could partly account for ethnic variations in A&G and referral rates.

We have split the graph in two with the rates of A&G and direct referrals by age and gender remaining in the manuscript as Figure 2, and adding rates of A&G and direct referrals by ethnicity and deprivation to the Supplementary Materials (Figure S1). We have not included the joint age×ethnicity analysis in the revised manuscript to remain within our approved protocol and to avoid small-cell disclosure risk in pre-pandemic years. The changes to the manuscript and Supplementary Materials are summarised below:

Results - *Rates of A&G by Socioeconomic Factors ~~Age, Gender, Ethnicity and Deprivation~~* (first sentence of each paragraph): Figure 2 depicts trends in prevalence of A&G and direct referrals over time stratified by age~~,~~ and gender, with Supplementary Figure S1 stratified by ethnicity~~,~~ and deprivation level. […]

White individuals had higher A&G prevalence than Asian, Black, and Mixed/Multiple ethnic groups (Supplementary Figure S1~~2~~). […]

Rates of Direct Referral by Socioeconomic Factors ~~Age, Gender, Ethnicity and Deprivation~~ (paragraph 2): Direct referrals decreased with increasing deprivation across ethnic groups in the years preceding the COVID-19 pandemic (2015-2019) and post pandemic (Supplementary 2022-2023; Figure S1~~2~~).). White patients had the highest referral prevalence, with the mixed/multiple ethnic group having the lowest rates. In 2020-2021 (lighter green dashed lines; Supplementary Figure S1~~2~~), referral rates dipped significantly, particularly among more deprived and ethnic minority groups (Supplementary Table S3).

Figure 2. Variation in A&G and referral use by age~~,~~ and gender~~, *deprivation, and ethnicity*~~ (2015 to 2023).

Caption: Distribution of A&G and direct referrals across ~~*key demographic and socioeconomic groups*~~ age and gender over time (2015 to 2023).

Supplementary Materials:

Figure S1 Variation in A&G and referral use by deprivation and ethnicity (2015 to 2023)

Discussion

7. Page 14, line 12: The discussion appropriately acknowledges that patient-important outcomes were not assessed. However, the later statement calling for a "redesign" of A&G may be too strong given that the study is descriptive and does not assess outcomes or cost-effectiveness. Consider softening this to recommend further evaluation before policy redesign is proposed.

We agree and have softened the language. We now recommend further evaluation of efficiency, equity and outcomes prior to policy redesign, while proposing targeted service improvements.

Discussion – *Conclusion* (last sentence): If A&G is to contribute meaningfully to the NHS Long-Term Plan to shift care into the community and reduce pressure on the elective care system,[25] ~~*it*~~ there

~~*must*~~ should be ~~*redesigned*~~ further evaluation and resourcing~~*ed*~~ to improve efficiency and equity, ensuring A&G does indeed ~~*it*~~ contribute~~*s*~~ to shorter, fairer, and more effective patient pathways.

Reviewer: 2 - Dr. Ismail Ismail, King's College London
Comments to the Author

Thank you for this interesting research on A&G and referral rates. It raises several important points about the variations in direct referral and A&G rates between different ethnic groups, and their trends over the years. With the recent push towards advice and guidance, this paper provides important data on its implementation and equity implications.
However in regards to your third aim, I have a couple of questions/comments about the methodology and results.

Methodology
1. How did you handle cases where a symptom could be mapped to several specialties?

2. It is unclear whether you only mapped 2023 cases where specialty was unclear, or applied this to all years (2015-2023). Could you clarify the scope of this mapping exercise?

3. You mentioned that the mapping algorithm was built using 2023 referral patterns - how did you account for evolving guidelines over the years, particularly if you applied this retrospectively to your study period?

We used frequency-based mapping. For each A&G record without a specialty recorded, we matched the nearest symptom/diagnosis code recorded 0–14 days prior to A&G to the most frequent specialty observed for that code in the random sample of individuals with a direct referral in 2023. Mapping was restricted to individuals with their first A&G record in 2023 only and not applied retrospectively therefore evolving guidelines were not specifically accounted for. We have made the following amendment to the *Mapping A&G Records* section of the **Methods** for clarity.

Methods - *Mapping A&G Records with Symptom/Diagnosis Codes*: A mapping exercise allowed target specialities to be determined using coded events in the 14 days prior to and on the date of an A&G record. The mapping exercise was restricted to A&G and direct referrals recorded in 2023 due to this being the most recently available year with the greatest number of A&G records available.

Results
4. Related to the second comment in the methodology subsection - if you did map all the years, then what were the findings for the earlier years? Did the proportion of mappable cases or specialty distribution change over time?

We specify in the *Mapping* section of the **Results** that we only included individuals with first A&G recorded in 2023, and believe the earlier clarification to the **Methods** addresses this.

Results – *Mapping* (first sentence): “Of 162,787 individuals recorded with their first A&G record in 2023, […]”

5. What happened to the 39% of the cases where a specialty could not be matched for 2023? Did this vary by ethnicity?

We did not propose any further analyses of individuals we could not determine specialties for via the mapping exercise in our approved CPRD protocol. We would be required to submit an amendment to the protocol to conduct this analysis. Whilst an interesting question, this is outside of our main objective to determine the feasibility of mapping potential specialties to A&G records.

Regarding your discussion

6. You stated that this is the first national population study - are there any comparisons to smaller or regional studies which could provide additional context?

We have added a theoretical framework for quality referrals, 2 exemplar specialty implementations in the *Comparison to Literature* section, and referenced an NHS England Referral Optimisation guideline for Dermatology. We note that while process gains (turnaround, triage, waiting times) are documented, robust evaluations of patient outcomes and cost-effectiveness are under-addressed remain a priority—particularly in practices serving disadvantaged populations.

Discussion – *Comparison to Literature* (paragraph 2): A scoping review defined four domains of referral quality: clinical information, clinical reasoning, patient factors, and system barriers.[22] Quality improved with the use of electronic templates, clear referral questions, guideline adherence, appropriate urgency, and documented preferences, while common deficits included missing histories/examinations and weak feedback loops. Exemplar evaluations of local specialty A&G implementation include:

(i) A&G embedded since 2017 supported ~6,500 patients' referral to Neurology in Newcastle -upon-Tyne over 2.5 years with a practical model for set-up, turnaround management and condition selection.[23] The authors reported efficiencies in triage and outlined barriers/solutions that other sites have since adopted;

(ii) an evaluation of >2,000 cases for general surgery in Birmingham showed A&G replies were typically same-day with a significant reduction in new outpatient appointments (16%) after A&G was introduced indicating improved triage and pre-clinic work-up;[24] and

(iii) national guidelines published by NHS England's Referral Optimisation for Skin Conditions and the British Association of Dermatologists e-RS A&G Frequently Asked Questions describe tele-dermatology A&G with rapid turnaround and image attachments to streamline referrals and mitigate inequities in access for inflammatory skin disease.[25]

References:

22. Osman S;, Harrison R;, Burton C;, Hider S;, Raiyat H;, Welsh V;, et al. Defining a theoretical framework for a quality referral at the primary-secondary care interface: a systematic scoping review with qualitative content analysis. British Journal of General Practice [Internet]. 2026 Jan 6 [cited 2026 Jan 6]; Available from: https://bjgp.org/content/early/2026/01/01/BJGP.2025.0304

23. Anderson KN, Warren N, Duddy M, McKean P, Miller JAL. Delivering an advice and guidance service in neurology. Pract Neurol [Internet]. 2022 Jun 1 [cited 2025 Dec 19];22(3):209–12. Available from: https://pn.bmj.com/content/22/3/209

24. Radnaeva I, Muthusami A, Ward S. NHS advice and guidance - improving outpatient flow and patient care in general surgery. Surgeon [Internet]. 2023 Mar 7 [cited 2025 Dec 19];21(5):e258–62. Available from: https://europepmc.org/article/MED/36894432

25. NHS England» Referral optimisation for people with skin conditions [Internet]. [cited 2025 Dec 19]. Available from: https://www.england.nhs.uk/long-read/referral-optimisation-for-people-with-skin-conditions/

7. You observed disparities but have not discussed possible reasons/mechanisms for this. I feel a brief discussion with existing literature may be useful to help contextualize your findings.

We have explored potential reasons and mechanisms for the disparities observed in our qualitative work, which we have described in response to comment 6 from reviewer 1. We have added findings from the literature to contextualise our findings in this manuscript:

Discussion – *Comparison to Literature* (paragraph 4): […] The Health Foundation demonstrated a deprivation gradient of those residing in more deprived areas experiencing poorer access and experience of primary care,[28] with the NHS Race and Health Observatory reporting higher levels of

discrimination and lower trust of primary care by Black and Asian patients.[29] The King's Fund subsequently conducted research into how these disparities were being addressed, highlighting barriers such as transport, employment constraints, and lower health literacy.[30] Our research suggests inequities ~~*may also*~~ arise ~~*earlier,*~~ at the point of referral itself, with A&G disproportionately applied to certain groups before a referral to secondary care is made. Our ongoing qualitative work will provide an in-depth analysis of patient and system factors that influence decision-making around mode of referral.

References:

28. Who gets in? - The Health Foundation [Internet]. [cited 2025 Dec 18]. Available from: https://www.health.org.uk/reports-and-analysis/analysis/who-gets-in

29. Patient Experience and Trust in NHS Primary Care - NHS – Race and Health Observatory [Internet]. [cited 2025 Dec 18]. Available from: https://nhsrho.org/research/patient-experience-and-trust-in-nhs-primary-care/

8. I do not think stating that A&G adds an additional step of unknown benefit is valid, as additional step seems to imply an unnecessary delay. There might be other reasons why a GP might A&G after referral, such as to ask for management advice whilst the patient waits to see the specialist. Without data on A&G content or patient outcomes, it may not be right to frame it the way as stated in the paper.

We agree with the reviewer and have amended to reflect on A&G often having direct referrals coded within ±4 months with further evaluation of the impact on efficiency/outcomes for patients required.

Discussion – *Comparison to Literature* (paragraph 3): However, the persistence of relatively high onward referral rates indicate~~*s*~~ that A&G may not be~~*is not yet*~~ fulfilling its intended role as a substitute for direct referral as they often co-occur; instead, ~~*it appears to add an additional step of unknown benefit in many*~~ the impact on efficiency and outcomes for patient journeys requires further evaluation.

9. You stated to support practices serving disadvantaged populations - do you have any suggestions on how to do this.

We have added suggestions using the framework for tackling inequalities published by NHS England in 2022.

Discussion – *Comparison to Literature* (last paragraph): […] NHS England (2022) published a practical framework to for NHS systems to tackle inequalities through actionable insights, which we believe could be implemented to support practices serving disadvantaged populations: (i) start with data (e.g., determine practices with greatest inequalities in patients receiving A&G/direct referral), (ii) test with lived experience (e.g., explore problems in receiving A&G/direct referral for those most in need), and (iii) co-design solutions with those most in need.[30]

References:

30. Tackling inequalities in healthcare access, experience, and outcomes Actionable Insights. [cited 2025 Dec 18]; Available from: https://www.england.nhs.uk/wp-content/uploads/2022/07/B1779-Actionable-Insights-Tackling-inequalities-in-healthcare-access-experience-and-outcomes-guidance-July-202.pdf

Reviewer: 3 - Matt Sutton, The University of Manchester
Comments to the Author
This manuscript uses a large sample of person-level records from the Clinical Practice Research Datalink to examine trends in the recording of 'advice and guidance' and 'referral to specialist' between 2015 and 2023. They also examine differences across population groups. There is interest in whether the two types of activity are occurring together and, if so, in what order and the paper

uncovers some differences in this between population groups.

I found the paper to be interesting and novel. Some comments that I think need to be addressed are below:

1. An easily understandable term should be used for “Advice & Guidance” which appears to be an English NHS term

2. The abstract needs a clear statement on why this is interesting. Why does it matter who gets “advice & guidance”? Why does it matter if this comes before or after referral? The text in the Conclusion suggests the authors have some concerns in mind (eg. efficiency, equity) but they don’t say so upfront

5. This sentence in the introduction is absolutely critical “Established in 2015, A&G is a two-way dialogue delivered synchronously (voice call) or asynchronously (electronic communication) that enables primary care to seek specialist input into a patient’s care through formal recorded means.” This is not at all clear from the label (I assumed it was A&G from the GP to the patient) and ‘remote specialist advice’ or something like that should be used throughout instead.

A&G is a term used extensively in clinical practice and healthcare policy and cannot be substituted. We have introduced A&G as accessing specialist advice remotely in the **Abstract**. We have added a clear statement in the **Abstract** foregrounding the efficiency and equity rationale for studying A&G and referral sequencing.

Abstract – Objective: Primary care remote access to specialist advice—known in the NHS as ‘Advice and Guidance’ (A&G)—is considered an important tool for managing waiting lists. However, there is limited evidence on how effective it is at reducing wider system pressures or ensuring equitable use. We aimed to examine trends and variation in the use of ~~*Advice and Guidance* (~~A&G~~)~~ compared with direct referrals in primary care, and to assess potential disparities across population groups.

3. Abstract should contain total population size

We have added the total population to the **Abstract** to address comment 1 from reviewer 1.

4. It doesn’t really make sense to talk a 19-fold increase when the baseline rate is so small. It is fine to say 1 in a 1000 people up to 20 in a 1000 people

We have changed references to 19-fold and 20-fold in the text to percentages with the amendments summarised in response to comment 3 from reviewer 1.

6. No justification is given for analysing whether or not an individual received an A&G or direct referral within a year, rather than the number of A&G or direct referrals within a year

To minimise the possibility of double counting recording of A&G requests/responses for the same clinical question, we felt it was more appropriate to model individuals recorded with A&G rather than the total number of A&G events. We have amended the Methods to reflect this.

Our decision was due, in part, to anticipated differences in coding between GP practices -- some practices only record A&G requests, some only record responses and some record both (based on the 4 codes we used to determine A&G records) -- and differential use of A&G nationwide (across practices, Trusts and commissioning localities). The introduction of Enhanced Service (£20 payment per episode of care for GP-initiated or GP-reviewed A&G) in March 2025 is beyond the scope of our study period (addressed in *Strengths & Limitations*, **Discussion**), but will influence clinical coding with incentivisation (as seen with ethnicity recording increasing during QOF incentivisation [2006-11] and decreasing after incentivisation withdrawal).

We have added differences in clinical coding and concern about true burden of A&G in primary care being unobserved to the *Strengths and Limitations* section of the **Discussion**.

Methods – *Statistical Analysis* (first sentence): We modelled annual person level prevalence (≥1 A&G/referral per year) to avoid doublecounting request/response entries and to minimise practice level coding heterogeneity. Annual person-level prevalence […]

Discussion – *Strengths and Limitations* (second sentence in second paragraph): Uptake of alternative tele-medicine providers may not be consistently coded in electronic health records meaning some specialist input is unobserved, while differences in clinical coding standards between practices (some only capturing A&G requests or responses rather than full pathway) and differential use of A&G across the healthcare system impacts our ability to infer the true burden of A&G on primary care.

7. Lines (38-29, page 5): I don't understand how region and commissioning locality could both be included as covariates. I assume one is nested in the other

8. No justification is given for including either region or commissioning locality or both as covariates

Thank you for spotting this oversight -- commissioning locality was used to stratify annual prevalence rates of A&G and direct referrals, but not included in the fully adjusted models. We have amended the *Statistical Analysis* section to reflect this.

Methods - *Statistical Analysis* (second sentence, third paragraph): Age, gender, region, ethnicity~~,~~ and deprivation ~~*and commissioning locality*~~ were included as covariates in adjusted models.

9. Differences in recording between practices would likely be important but practice is not included as one of the covariates. We therefore do not know whether the differences by population subgroup are due to within- or between-practice differences.

Thank you for identifying this omission – robust standard errors clustered at practice level were calculated for all models. We have added this to the *Statistical Analysis* section.

Results – *Statistical Analysis* (third paragraph, last sentence): All models included robust standard errors clustered at the practice level.

10. I was not clear how the denominator population has been defined. The figure given is 16.3million, which sounds like a snapshot for one point in time rather than repeated value for every year. Some patients will join and some will leave the registered population over the period so it is unclear how the prevalent rates in Table 1 are calculated

Patients can join and leave the annual denominator population across the study period. The figure of 16.3 million is the total number of individuals ever contributing to an annual denominator population. We have added to the **Results** for clarity.

Results (first sentence): We analysed data on 16.3 million registered patients who were included in the annual denominator population at any point during the study period.

11. This sentence (lines 55-56 page 8) is critical: "This challenges the assumption that A&G typically precedes referral." This needs to come before the analysis is presented to explain why the work is being conducted. It is not obvious to the reader on first reading. While the rates of use of A&G and direct referral are interesting, I think the analysis of whether they both happen and, if so, in which order, is a more interesting line of enquiry.

We agree, and have added to the **Introduction** to foreground our analysis of direct referrals recorded within ±4 months of A&G.

Introduction (first sentence, paragraph 4): However, it is unclear ~~*how and*~~ which patient groups A&G is currently used for, whether A&G is an adjunct to direct referral and the order of elective care

requests (e.g. A&G before, at the time of, or after direct referral), and whether there is variation in ~~its~~ use of A&G by sociodemographic characteristics.

Reviewer: 4 - Prof. Katie Harron
Comments to the Author
This is an interesting description of the use of A&G and direct referrals in primary care. I have a few points for clarification.

1. Firstly, I think there needs to be a more clear and upfront description of what A&G is, and why it might be used (with and without referral). A clinical example here would be helpful.

We agree and have added a sentence in the **Abstract** to address comment 2 from reviewer 3.

2. Abstract: please give the total number of individuals/person-years included

We have added the total population to the **Abstract** to address comment 1 from reviewer 1.

3. Given the authors are interested in trends in the use of A&G, I think it would make more sense to describe and model the total number of A&G events per person-year, rather than the proportion of patients with at least one A&G event within a given year. What were trends in the total numbers of A&G like?

We believe we have addressed this in response to comment 6 from reviewer 3 with anticipated differences in coding between practices, and concerns about double-counting requests and responses for the same problem.

4. How has the number of individuals who were assigned A&G alone, compared with both A&G and direct referral, changed over time?
These findings are summarised in Table 3 and we have added the trends over time to the **Results**.

Results - *Proportion of Individuals with A&G and Direct Referrals Recorded* (second sentence): The proportion of individuals with A&G only was marginally higher between 2015-2017 (17%-18%) before stabilising between 13%-14% in more recent years (2018-2023; Table 3).

5. The multinomial modelling needs more explanation and description. Was this a single model, with direct referral before / direct referral after / no direct referral as the outcome? Or were separate models used?
We have added the comparison groups to the *Statistical Analysis* section of the **Methods**.

Results – *Statistical Analysis* (third paragraph): The percentage of individuals recorded with A&G also recorded with a direct referral in the 4 months (122 days; agreed by clinical consensus) i) before (-122 to 0 days) and ii) after (1 to 122 days) A&G record were derived and compared by the covariates (excluding those with less than four months of follow-up), descriptively and by using multinomial logistic regression to compare those with A&G only to those with direct referral recorded before A&G and those recorded with direct referral after A&G […]

6. How was clustering of observations within practices dealt with?

Thank you for identifying this omission. We have addressed this in response to comment 9 from reviewer 3 by adding to the **Methods** that robust standard errors clustered at practice level were calculated for all models.

7. Fig2: The text states that "Although A&G is more prevalent in males over time, a higher percentage of females were recorded with A&G due to males experiencing more episodes" I don't understand this

explanation. The plot is showing percentages– so relative comparisons shouldn't be affected by the total number of episodes. Table 1 also indicates that prevalence is higher in females (not males).

Thank you for spotting this error. Males and Females were accurately labelled in Table 1 (A&G only) and Table 3, but mislabelled in Figure 2 (not shown below), Table 1 (direct referrals only), Table 2 and Supplementary Materials, Table S2 and Table S3 (neither shown below). We have itemised the changes to the manuscript and affected tables/figures below:

Results – *Rates of A&G by Socioeconomic Factors* (second sentence): ~~*Although*~~ A&G is more prevalent in females over time~~*, a higher percentage of females were recorded with A&G due to males experiencing more episodes*~~.

Results – *Rates of Direct Referral by Socioeconomic Factors* (first sentence): Direct referrals increased with age from ~~2~~15 years in females and ~~1~~25 years in males, peaking in those aged 75–84 years, with slightly higher referral prevalence in females than ~~fe~~males for most age groups (Figure 2).

Table 1:

Gender	Females	8,210,533	395,228 (4.8)	5,226,933 (64.3) ~~4,492,863 (54.7)~~
	Males	8,130,163	276,666 (3.4)	4,492,863 (54.7) ~~5,226,933 (64.3)~~

Table 2:

Gender	Females	2.40 (2.38-2.41) ~~1.56 (1.55-1.57)~~	27.6 (27.6-27.7) ~~19.7 (19.7-19.7)~~
	Males	1.56 (1.55-1.57) ~~2.40 (2.38-2.41)~~	19.7 (19.7-19.7) ~~27.6 (27.6-27.7)~~

8. The annual prevalences in table 2 are presented differently to the overall prevalence in Table 1, i.e. as a rate rather than a percentage. Have these been calculated differently or do they represent the same thing, but for different periods? Table 2 is labelled as prevalence rate %, which is unclear.

We thank the reviewer for pointing out this discrepancy. We have calculated prevalence consistently in our analyses as the percentage of individuals in a given calendar year with A&G or direct referral recorded divided by the registered population for the same calendar year. We have removed reference to rates from the *Statistical Analysis* section and from Table 2.

Methods - *Statistical Analysis* (second sentence): Annual person-level prevalence ~~*rates*~~ for A&G, direct referrals and NHS e-RS Open Data Dashboard were calculated as a percentage of the registered population having at least 1 A&G (or referral, as appropriate) recorded during the year.

Table 2 (first cell): Prevalence ~~*rate*~~, % (95% CI)

9. Table 3- which period is this for? Given the unadjusted and adjusted results are similar, and given there is a lot going on in this table, I suggest keeping one set of results here and putting the rest in an abstract. I think that would make the comparisons (before / after; between demographic groups) easier to digest. The adjusted results I think are adjusted for CCG but the coefficients are not presented. What was variation by CCG like?

The analyses presented in Table 3 were conducted over the full study period (2015-2023) and we have added this to the table title. We did not include commissioning locality as a covariate in the analysis due to it being embedded within geographical region. We have amended the *Statistical Analysis* section to reflect this in response to comments 7-8 from reviewer 3.

Table 3 Association between A&G and Direct Referral within ±4 months (2015-2023)

10. Fig 3 – what do the numbers along the x axis refer to?

We were provided with de-identified commissioning localities and the numbers on the x-axis correspond to a label from 1 to n for commissioning localities within each region, ordered on the

percentage of individuals recorded with A&G from lowest to highest. We have added a footnote to the Figure 3 caption to explain the numbering.

Figure 3 – caption: Commissioning localities within each region are numbered from smallest to largest percentage of individuals recorded with A&G in that locality.

11. Table S2 etc– need to state what the numbers in the parentheses refer to.

Thank you for spotting this omission – we have added “95% confidence interval” to the table caption.

Supplementary Materials:

Table S2 Prevalence of A&G by Sociodemographic Factors and Year, % (95% confidence interval)

Table S3 Prevalence of Direct Referrals by Sociodemographic Factors and Year, % (95% confidence interval)

VERSION 2 – REVIEW

**REVIEWER NAME**	Ismail, Ismail
**REVIEWER AFFILIATION**	King's College London, Department of Population Health Sciences
**REVIEWER CONFLICT OF INTEREST**	None
**AI DISCLOSURE**	No
**DATE REVIEW RETURNED**	04-Feb-2026

**GENERAL COMMENTS**	This is a revision. I feel the authors have sufficiently addressed the comments raised and I have no further revisions to suggest.

**REVIEWER NAME**	Harron, Katie
**REVIEWER AFFILIATION**	
**REVIEWER CONFLICT OF INTEREST**	None
**AI DISCLOSURE**	No
**DATE REVIEW RETURNED**	19-Jan-2026

**GENERAL COMMENTS**	The authors have addressed my previous comments and I don't have anything else to add.
